# Advice Network Centrality as a Social Origin of Task Crafting: The Bridging Roles of Basic Psychological Needs

**DOI:** 10.3390/bs14060440

**Published:** 2024-05-24

**Authors:** Inyong Shin

**Affiliations:** Division of Business Administration, Pukyong National University, Busan 48513, Republic of Korea; shiny@pknu.ac.kr; Tel.: +82-51-629-5726

**Keywords:** advice network centrality, basic psychological needs, task crafting

## Abstract

Little is known about the predictive role of advice networks in task crafting despite the growing academic and practical interest in its antecedents. Accordingly, as centrality in advice networks is expected to have a positive relationship with task crafting, this study develops a research model encompassing the mediating roles of the fulfillment of basic psychological needs to clarify this relationship. The model was tested using a sample composed of 198 employees from various firms in South Korea. The results showed that employees who occupy central positions in the advice network fulfilled their autonomy and competence needs, consequently engaging in task crafting. This study contributes to the literature on social networks, self-determination, and task crafting by discovering hidden antecedents and pivotal mechanisms in determining task crafting.

## 1. Introduction

The expansion of empowerment, advances in information technology, and the proliferation of telecommuting have increased employees’ autonomy over their work and, thus, their interest in shaping their roles [1,2,3]. In this situation, employees often take actions to redefine and redesign their jobs, captured by the concept of job crafting [4]. Furthermore, the attention of organizations is now shifting from top-down interventions aiming to design jobs in advance to bottom-up interventions based on job crafting [5]. In particular, task crafting, which indicates the self-initiated behavior of proactively altering the number, scope, or type of tasks carried out at work [4], is acknowledged as the core of job crafting. Evidence for this has accumulated, indicating that employees who craft tasks tend to have improved health and well-being and contribute significantly to their organizations [6,7,8].

Researchers have recognized the importance and effectiveness of task crafting and thus have made efforts to uncover the factors promoting it. Prior studies on the antecedents of task crafting have largely focused on individual characteristics, such as openness to experience and career orientation, as well as job characteristics, including work discretion and job autonomy [6,9,10,11]. These studies contribute to the task crafting literature by broadening the understanding of who becomes task crafters in which work contexts.

However, social contexts within organizations have been neglected in the research on discovering the causes of task crafting. Under the acknowledgment that jobs and roles are embedded in social structures, interpersonal relations occurring at work, which serve as opportunities to guide individual behavior, are increasingly viewed as valuable. From a social network perspective, the nature of relational patterns can generate specific conditions for obtaining access to key organizational resources that determine individual behavior [12,13]. In addition, social networks reflect the quantity and quality of actual workplace relations, allowing us to understand relational patterns in detail [14]. Various social networks coexist in the workplace, distinguished by the contents transmitted through ties or connections [12]. Among them, advice networks have received substantial scholarly attention. These networks refer to the relational patterns through which employees share resources related to task completion, including information, assistance, and guidance [15,16,17]. Compared to employees located on the periphery of advice networks, those in the center have greater social reach and easier access to resources, leading to higher levels of performance and innovation [16,17]. In addition, it has been reported that central positions in advice networks are positively associated with intentional actions to generate and realize new ideas within an organization, as well as constructive change-oriented communication to improve situations [18]. Accordingly, with the expectation that employees’ central positions in their advice networks are likely to affect their behavior in changing task boundaries, this study seeks to clearly explain the link between advice network centrality and task crafting.

Authors who have developed the model of job crafting suggest individual needs as key factors [4]. This study explores employees’ needs, drawing on the self-determination theory, which is considered an appropriate framework for understanding individual needs [19,20,21]. The theory assumes that the natural tendency for individual development is directed toward psychological growth, internalization, and well-being, and that the fulfillment of basic psychological needs (i.e., the needs for autonomy, competence, and relatedness) is essential to achieve them. Previous studies have revealed that satisfying basic psychological needs promotes health, attitudes, performance, and creativity (e.g., [22,23,24]). Meanwhile, workers’ social contexts within organizations, which are a means of fulfilling needs [25], have been recognized as potential antecedents of job crafting [4]. However, research has rarely been conducted on whether the social environment of an organization, embodied in an advice network, affects the satisfaction of organizational members’ basic psychological needs, and if the satisfaction of those needs influences task crafting. Thus, this study strives to address this research gap by examining the mediating roles of the basic psychological needs in the relationship between advice network centrality and task crafting.

Overall, this study seeks to explore whether advice network centrality relates to the fulfillment of basic psychological needs, which in turn relates to task crafting. The next section proposes hypotheses regarding these relationships based on theories of social networks, self-determination, and job crafting. The data collection, construct measures, and empirical results are then described in detail. Finally, the findings of this study are discussed in terms of the theoretical contributions and practical implications.

This study is expected to make important contributions in three aspects. First, it identifies that advice network centrality serves as the social origin of task crafting, expanding the research on task crafting. Second, it validates how fulfilling each of the three basic psychological needs links advice network centrality to task crafting, elaborating on the self-determination theory. Lastly, it explores the effectiveness of advice networks around motivational states, which contributes to the social network literature.

## 2. Theoretical Review and Hypothesis Development

### 2.1. Advice Networks and Need Fulfillment

Employees embedded in advice networks share resources by communicating organizational issues [16,17]. In advice networks, some employees are often sought after by their colleagues for these resources. When investigating the effect of intra-organizational networks on individual behavior, an employee’s position in a network typically draws attention, which is conceptualized as centrality in the network, representing the number of ties between a focal person and others [26]. In particular, the most widely used type of network centrality is indegree centrality, which indicates an individual’s activity, popularity, or prominence [27]. Accordingly, occupying central positions in the advice network provides the occupants with social reach and prestige [12]. As a result, employees in the center are more likely to perform assigned tasks and engage in innovative activities than those in the periphery [16,17,18].

The self-determination theory is a motivational framework based on the assumption that individuals have basic psychological needs for personal growth and that fulfilling those needs is an essential prerequisite for their flourishing and development [19,20,21]. Unlike related theories, which are concerned with stable individual differences in need intensity, this theory emphasizes differences in opportunities to fulfill needs. This study examines whether the degree of centrality in an advice network is a prerequisite for satisfying three basic psychological needs—autonomy, competence, and relatedness.

In the work context, autonomy refers to the feeling of being able to control one’s work environment and self-organize his or her behavior by experiencing the volition and authenticity of his or her work-related thoughts and actions [19,20]. Notably, the need for autonomy does not mean that one should act independently of others’ desires, but rather, one should act with choice and will, even if it is interpreted as following the wishes of others. Meanwhile, as indegree centrality in the advice network provides others with a good proxy for expertise [28], others tend to rely on individuals in central network positions to obtain relevant resources. Their power increases as others depend on them [27,28]. In addition, those who hold central positions in advice networks enjoy greater informal influence due to their social reach and reputation [29,30]. As their power and influence over colleagues asking for information and advice increases, so does their autonomy. Indeed, it has been revealed that advice network centrality and autonomy at work are correlated [31]. As a result, they are likely to take control of their work environment, organize their work behaviors, and even enjoy increased social status. Therefore, employees who frequently interact with their colleagues in terms of work-related advice are likely to have their needs for autonomy met.

Competence refers to the feeling of being skillful and effective. The need for competence at work is seen as the need to feel a sense of effectiveness and mastery [19,20]. The need for competence, derived from opportunities to apply and expand abilities, implies a natural tendency to explore and manipulate one’s surroundings to pursue challenges. The role of an advice network in satisfying the need for competence stems from enabling individuals embedded in the network to engage in and solve work-related challenges and problems. Employees who give information and advice to others regarding work may have a better understanding of the nature, causes, and alternatives of work-related problems [32]. Advice networks also allow central employees to gain unique insights into current issues in various organizational aspects [33]. Further, employees who occupy central positions in the advice network are likely to be recognized as informal leaders and to have a high level of leader–member exchange with formal leaders [34]. This makes it easy for them to obtain feedback related to performance and roles from their formal leaders [35]. These benefits ensure that individuals who occupy central positions in the advice network are more likely to become proficient at their work, thereby generating more feasible and higher-quality solutions to work problems [36,37]. Therefore, central employees in the advice network have the potential to satisfy their competence needs.

Relatedness refers to the feeling of connectedness and unity and includes a sense of being important to others. It denotes experiences of warmth, caring, and bonding, including a sense of being connected to significant others, having caring relationships, and belonging to a work community [19,20]. The need for relatedness indicates the desire to feel connected to others, namely the need to care for others, and to be cared for in return. The need for relatedness in the workplace is satisfied when organizational members form and maintain close relationships, view themselves as organizational members, and experience a sense of solidarity. If coworkers frequently ask an employee for advice, the employee can feel meaningful and helpful within the organization, leading him or her to develop a sense of belonging. It has been also found that the number of advice ties employees maintain is positively related to their feelings of attachment and inclusion at work [38]. In addition, asking an individual for advice within an organization means respecting the individual’s opinion and indicates the expectation that the individual’s help is useful. As a result, employees who occupy central positions in the advice network gain trust by providing reliable information and advice to their colleagues [39]. Therefore, advice network centrality is likely to be an important source of relatedness need satisfaction.

Based on the above discussion, this study expects that employees who are centrally located in an advice network will satisfy their needs for autonomy, competence, and relatedness. Therefore, the following hypotheses are suggested:

**Hypothesis 1a.**  *Advice network centrality will be positively related to autonomy need fulfillment*.

**Hypothesis 1b.**  *Advice network centrality will be positively related to competence need fulfillment*.

**Hypothesis 1c.**  *Advice network centrality will be positively related to relatedness need fulfillment*.

### 2.2. Need Fulfillment and Task Crafting

Although no prior research has empirically examined the link between the satisfaction of basic psychological needs and task crafting, this study posits that the satisfaction of those needs has ample potential to promote task crafting. According to the self-determination theory, basic psychological needs represent essential energy sources for optimal functioning. Satisfying these needs determines the extent to which individuals make their work-related activities more meaningful and proactive [21].

Specifically, perceived opportunities are necessary conditions to lead to job crafting [4], and one of the factors that promote such opportunities is the sense of autonomy employees have in what they undertake in their tasks and how they execute them [40]. When employees are given discretion in how to pursue and achieve their work, they are inclined to craft their tasks [10,11]. Next, workers with a sense of competence tend to set tougher standards and goals for themselves, find opportunities to demonstrate their abilities, and pay attention to developmental possibilities [41]. Lastly, changing one’s task boundaries on his or her own may put others at risk by jeopardizing their capacities to perform the tasks involved [4]. The state in which the need for relatedness of an employee is satisfied, based on the trust he or she has gained from his or her colleagues, provides the employee with a safe context for task-related challenges and risk-taking work behavior [42].

Further, it is noteworthy that fulfilling these needs autonomously motivates individuals. When employees are in a motivational state that allows them to be interested and participate in their jobs or internalize the importance and value of the work, they naturally become immersed in tasks and exert considerable effort [21,43]. Such a state allows employees to withstand obstacles when performing tasks and increases their activities and initiative [44]. Therefore, they are more inclined to challenge themselves by voluntarily asking for more responsibilities, taking on new approaches, or altering procedures [45,46].

In sum, it is expected that employees whose needs for autonomy, competence, and relatedness are satisfied will exhibit proactive behavior to change their tasks. Thus, this study proposes the following hypotheses:

**Hypothesis 2a.**  *Autonomy need fulfillment will be positively related to task crafting*.

**Hypothesis 2b.**  *Competence need fulfillment will be positively related to task crafting*.

**Hypothesis 2c.**  *Relatedness need fulfillment will be positively related to task crafting*.

### 2.3. Advice Networks, Need Fulfillment, and Task Crafting

Based on the rationale discussed in the previous sections, this study predicts that employees who occupy central positions in the advice network will fulfill their needs for autonomy, competence, and relatedness. Furthermore, those who have satisfied their basic psychological needs have greater potential to voluntarily change the boundaries of their tasks. The integration of these predictions implies that the extent to which employees are involved in task crafting is determined by the fulfillment of their basic psychological needs, which is facilitated by their central positions in the advice network. In other words, a distal antecedent of task crafting is advice network centrality, and its proximal antecedents are the fulfillment of basic psychological needs. Thus, this study proposes that employees high in advice network centrality will craft their tasks by fulfilling their basic psychological needs. Therefore, the following hypotheses are suggested:

**Hypothesis 3a.**  *Advice network centrality will be positively and indirectly related to task crafting via autonomy need fulfillment*.

**Hypothesis 3b.**  *Advice network centrality will be positively and indirectly related to task crafting via competence need fulfillment*.

**Hypothesis 3c.**  *Advice network centrality will be positively and indirectly related to task crafting via relatedness need fulfillment*.

**Figure 1:**
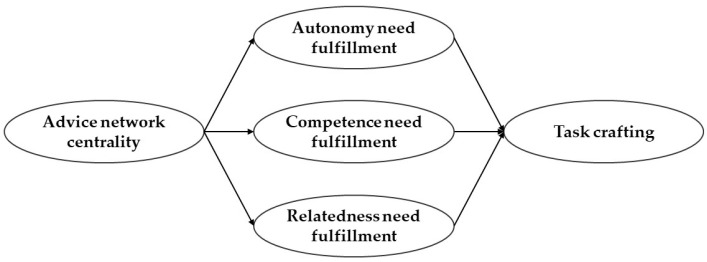
Conceptual model.

Figure 1 presents the conceptual model, which synthesizes the hypotheses included in this study.

## 3. Method

### 3.1. Sample and Procedure

Data were collected from employees working for various small and medium-sized firms (i.e., education, telecommunications, banking, and high-tech companies) across industries in South Korea to identify whether the hypotheses presented in this study were supported regardless of firm-specific characteristics. To obtain data, the author first met with each company’s management team and human resource manager and explained the purpose and content of the survey. Then, the author confirmed the participants’ consent to participate in the survey, provided them with the questionnaires, and collected them after they were filled out. The questionnaires were distributed to 208 employees, and 198 returned them (a response rate of 95.2%).

The final sample was 198 full-time employees, consisting of 72 employees in the manufacturing industry (36.4%) and 126 employees in the service industry (63.6%). On average, each company had 33.5 employees. The gender distribution comprised 123 men (62.1%) and 75 women (37.9%), and the average organizational tenure was 5.4 years.

### 3.2. Measures

#### 3.2.1. Advice Network Centrality

This study employed a social network survey to identify advice network centrality. It used the roster method to list the names of all employees in an organization to obtain accurate and objective responses [14,47]. The respondents were asked to go through a list and check the names of employees to whom they go for advice and help about work-related issues. This study entered these responses into UCINET 6 to calculate indegree centrality in the advice network, which refers to the total number of employees who nominated the focal person as their source of advice [26,48]. Indegree centrality was adopted as an appropriate indicator for this study because it overcomes the limitations of the self-report method [17,49]. Indegree centrality in the advice network is derived from the number of ties that seek advice from specific employees, indicating that employees with higher centrality in the advice network offer more advice than those with lower centrality. Finally, to reduce the bias caused by different sizes of networks and to enable the comparison of the derived centrality values across the networks, each centrality value was divided by n – 1 (n = network size) and multiplied by 100 to transform it into a normalized value from 0 to 100 [14,50].

#### 3.2.2. Basic Psychological Needs

This study measured basic psychological needs using the Work-related Basic Need Satisfaction Scale by Van den Broeck and his colleagues [23]. Specifically, each need was measured using a 4-item scale and with a five-point response scale (1 = strongly disagree to 5 = strongly agree). The sample items are “I feel like I can be myself at my job (autonomy need fulfillment)”, “I really master my tasks at my job (competence need fulfillment)”, and “I do not really feel connected with other people at my job (relatedness need fulfillment; reverse coded)”. The Cronbach’s alpha value was 0.72 for autonomy need fulfillment, 0.87 for competence need fulfillment, and 0.80 for relatedness need fulfillment.

#### 3.2.3. Task Crafting

Task crafting was measured using Slemp and Vella-Brodrick’s [51] 6-item scale with a five-point response scale (1 = strongly disagree to 5 = strongly agree). A sample item is “I change the scope or types of tasks on my own”. The Cronbach’s alpha value was 0.92.

#### 3.2.4. Control Variables

Given that the respondents who participated in the survey belonged to several organizations in different industries, industry type (0 = manufacturing, 1 = service) and organization size (the number of employees) were controlled. In addition, prior research has shown that gender and organizational tenure are associated with task crafting (e.g., [7,52]). Accordingly, this study controlled for individual differences, in gender (0 = female, 1 = male) and organizational tenure (in years). Lastly, openness to experience has been found to have a positive relationship with task crafting [9]. Thus, this study measured openness to experience using four items corresponding to the construct from the International Personality Item Pool [53] with a five-point response scale (1 = strongly disagree to 5 = strongly agree). The Cronbach’s alpha value for openness to experience was 0.83.

## 4. Results

### 4.1. Reliability and Validity Testing

The Cronbach’s alpha values for the psychometric variables included in this study (i.e., basic psychological needs, task crafting, and openness to experience) were found to have satisfactory internal consistency, ranging from 0.72 to 0.92 [54]. A confirmatory factor analysis was conducted using AMOS 21 to evaluate the variables’ convergent and discriminant validity. As a result of this analysis, the measurement model indicated an acceptable fit (χ^2^_(176)_ = 348.70, CFI [comparative fit index] = 0.92; TLI [Tucker–Lewis index] = 0.91; RMSEA [root mean square error of approximation] = 0.07; SRMR [standardized root mean square residual] = 0.07). Each item’s standardized factor loading value ranged from 0.58 to 0.92, and the composite reliability of all variables ranged from 0.80 to 0.94. Each variable’s average variance extracted value fell between 0.58 and 0.75 and was larger than the squared values of the correlation coefficients between the variables. As a result, the variables were judged to have acceptable levels of convergent and discriminant validity (cf. [54,55]).

### 4.2. Hypothesis Testing

Table 1 contains the means and standard deviations of this study’s variables, as well as the correlations among them. This study used SPSS (version 27.0) to determine whether Hypotheses 1a–1c and 2a–2c were supported. Table 2 presents the standardized coefficients derived using regressing advice network centrality with the three basic psychological needs as mediators and task crafting as the dependent variable.

Hypotheses 1a–1c stated that employees centrally located in the advice network would fulfill their autonomy, competence, and relatedness needs. As shown in Table 2, the results showed that while advice network centrality was positively and significantly related to the fulfillment of autonomy (Model 1: *β* = 0.22, *p* < 0.01) and competence needs (Model 2: *β* = 0.15, *p* < 0.05), it was not significantly related to the fulfillment of relatedness needs (Model 3: *β* = 0.10, n.s.). As a result, Hypotheses 1a and 1b were supported, but Hypothesis 1c was not.

Hypotheses 2a–2c predicted that fulfilling the three basic psychological needs would be positively associated with task crafting. As indicated in Table 2, autonomy (Model 4: *β* = 0.34, *p* < 0.01) and competence need fulfillment (Model 4: *β* = 0.24, *p* < 0.01) were found to be positively and significantly related to task crafting. However, relatedness need fulfillment was not related (Model 4: *β* = −0.04, n.s.). Thus, Hypotheses 2a and 2b were supported, but Hypothesis 2c was not.

Hypotheses 3a–3c proposed that employees high in advice network centrality would have their needs for autonomy, competence, and relatedness fulfilled, thereby leading them to engage in task crafting. This study used the multiple mediator model 4 of PROCESS macro (version 3.5) program [56] to determine whether Hypotheses 3a–3c were supported. As a measure of the indirect effect in this study, the bias-corrected bootstrap with 95% confidence intervals (CI) was obtained using a total of 5000 bootstrap samples. In the absence of zero included in the 95% CI, the indirect effect was considered significant. As seen in Table 3, the indirect effects of advice network centrality on task crafting via the fulfillment of autonomy (*b* = 0.004, CI = [0.001, 0.007]) and competence needs (*b* = 0.002, CI = [0.000, 0.004]) were positive and significant. However, the indirect effect via relatedness need fulfillment was not significant (*b* = −0.000, CI [−0.001, 0.001]). The total indirect effect was statistically significant (*b* = 0.006, CI = [0.002, 0.009]). Therefore, Hypotheses 3a and 3b were supported, but Hypothesis 3c was not. In addition, the direct effect of advice network centrality on task crafting was still statistically significant (*b* = 0.007, CI = [0.001, 0.013]). In sum, this study confirmed that the positive relationship between advice network centrality and task crafting was partially mediated by the fulfillment of autonomy and competence needs.

## 5. Discussion

### 5.1. Overall Findings

This study attempted to identify whether centrality in the advice network has a positive relationship with task crafting by fulfilling basic psychological needs. As a result of analyzing the data obtained from surveying 198 employees working from various companies in South Korea, it was revealed that employees central to the advice network tended to have their needs for autonomy and competence met, consequently leading them to engage in task crafting. These findings imply that the distal antecedent of task crafting is advice network centrality, and the proximal antecedents are the fulfillment of autonomy and competence needs.

However, it was also found that fulfilling the need for relatedness did not link the relationship between advice network centrality and task crafting. This is interpreted partly because advice networks contain instrumental characteristics for exchanging information and obtaining advice [16], which may make the actors embedded in the networks feel less of a sense of belonging and care. Furthermore, given that the need for relatedness is often characterized as less immediately imperative for some outcomes than the other needs [22,23], satisfying this need does not appear to directly affect task crafting.

### 5.2. Theoretical Contributions

The findings of this study make several contributions to the relevant literature. First, this study helps expand the task crafting research by indicating that advice network centrality serves as the social origin of task crafting. It has been proposed that social contexts, such as interpersonal relations at work, are important in understanding task crafting at the individual level [40,57], but little effort has been made to account for the roles of relational patterns in determining task crafting by adopting a social network perspective. This seems to be because most researchers take a more agentic view toward employees. This study has demonstrated the feasibility of advice networks as social sources that promote task crafting among employees. Therefore, it has been revealed that social characteristics act as antecedents of task crafting beyond the individual and job characteristics focused on in existing studies (e.g., [6,9,10,11]).

Second, this study contributes to the elaboration of the self-determination theory by validating how fulfilling each of the three basic psychological needs links advice network centrality to task crafting. The main principle of the self-determination theory is that social factors satisfy basic psychological needs, which energizes motivational states that lead to adaptive outcomes [19,58]. Therefore, the findings of this study, that employees change their tasks by satisfying their basic psychological needs, not to satisfy those needs, are consistent with the original premise of the self-determination theory. This theory also posits that satisfying specific needs is a function of the social conditions under which employees perform their jobs and roles [58] and that it is important to focus on each need to understand their differential relationships with specific outcomes [22]. By revealing that the relationship between advice network centrality and task crafting is partially mediated by the fulfillment of autonomy and competence needs, this study identifies that the prerequisites for satisfying needs, and the effects after their satisfaction, are differentiated depending on each need.

Lastly, this study contributes to the social network literature by exploring the effectiveness of social networks around motivational states. Noting that studies on social networks have largely assumed that motivational states are endogenous to network structures (e.g., [59,60]), some scholars have suggested that much remains to be done to determine how network characteristics affect individual motivation [61]. Thus, this study addresses this research gap by demonstrating that the extent to which employees are embedded in advice networks contributes to satisfying their psychological needs for being autonomously motivated.

### 5.3. Practical Implications

This study provides several practical implications for managers to encourage employees to engage in task crafting. Specifically, prior work has revealed that high self-monitors and conscientious individuals tend to be the targets of colleagues seeking advice, consequently positioning them at the center of advice networks [37]. Accordingly, identifying the personality characteristics of employees can infer who plays key roles in advice networks. Then, by examining informal relations within the organization, it is necessary to identify which person is located at the center [62]. Further, it is necessary to establish a horizontal organizational culture to exchange information and advice smoothly. These efforts will likely encourage organizational members to participate more in task crafting and help promote knowledge sharing and social support.

To encourage employees to change their task boundaries immediately, satisfying their autonomy and competence needs is desirable. Transformational leadership is known to play a key role in meeting these needs [25]. Accordingly, employees’ autonomy and competence needs will likely be met if managers provide ideal influence, inspirational motivation, individualized consideration, and intellectual stimulation.

### 5.4. Limitations and Future Research Directions

Although this study provides various contributions and implications, it also has some limitations. First, due to the relatively small sample size, this study conducted regression analyses to identify the relationships between variables. If data from more diverse organizations and more employees are collected and the relationships between variables are simultaneously estimated with the structural equation modeling, more statistically valid results would be obtained.

Second, it is necessary to examine the causal relationship between the fulfillment of basic psychological needs and task crafting. Although this study developed and tested the model that the fulfillment of basic psychological needs would have a positive relationship with task crafting, some scholars proposed that task crafting could influence the satisfaction of the needs (e.g., [63,64]). It is expected that future studies will closely examine the relationship between need satisfaction and task crafting based on a longitudinal research design.

Third, the findings of this study can be advanced through the additional consideration of individual characteristics in how advice networks influence task crafting. That is, researchers should study the combination of advice networks and personalities that influence task crafting. For example, if proactive employees occupy central positions in the advice network, they will likely engage in task crafting more actively. This remains a topic for future research.

Finally, it is necessary to expand this study, which attempted to discover the antecedents of task crafting in indegree centrality in advice networks. Focusing on the aspect that indegree centrality in advice networks represents advice-giving, this study found that advice network centrality is related to the fulfillment of the needs for autonomy and competence, but it overlooked the roles of other types of networks and centrality. For instance, indegree centrality in friendship networks may help satisfy the need for relatedness by gaining emotion-based trust and social support, as well as outdegree centrality in advice networks, meaning advice-receiving, which may contribute to satisfying the need for competence. It is hoped that future research will reveal how different types of networks and centrality affect the satisfaction of basic psychological needs.

## Figures and Tables

**Table 1:** Means, standard deviations, and correlations.

Variables	Mean	SD	1	2	3	4	5	6	7	8	9
1. Industry	0.64	0.48									
2. Organization size	33.53	10.45	−0.44 *								
3. Gender	0.62	0.49	0.15 *	0.04							
4. Organizational tenure	5.42	6.42	0.12	0.23 **	0.02						
5. Openness to experience	3.31	0.53	0.20 **	−0.16 *	0.16 *	0.02					
6. Advice network centrality	14.01	13.18	0.35 **	−0.32 **	0.22 **	0.15 *	0.22 **				
7. Autonomy need fulfillment	3.19	0.69	0.07	0.06	0.23 **	0.09	0.25 **	0.25 **			
8. Competence need fulfillment	3.48	0.68	0.18 **	0.02	0.24 **	0.14 *	0.39 **	0.25 **	0.44 **		
9. Relatedness need fulfillment	3.75	0.68	0.20 **	−0.03	0.17 *	0.08	0.13	0.17 *	0.21 **	0.39 **	
10. Task crafting	3.32	0.68	0.11	−0.02	0.29 **	0.22 **	0.43 **	0.35 **	0.55 **	0.52 **	0.19 **

Note. *N* = 198. All tests are two-tailed, * *p* < 0.05, ** *p* < 0.01.

**Table 2:** Regression results for basic psychological needs and task crafting.

Variables	Autonomy Need Fulfillment	Competence Need Fulfillment	Relatedness Need Fulfillment	Task Crafting
	Model 1	Model 2	Model 3	Model 4
Industry	−0.01	0.10	0.16	−0.10
Organization size	0.15	0.15	0.08	−0.05
Gender	0.14 *	0.13	0.11	0.11
Organizational tenure	0.01	0.06	0.03	0.15 **
Openness to experience	0.21 **	0.34 **	0.07	0.22 **
Advice network centrality	0.22 **	0.15 *	0.10	0.13 *
Autonomy need fulfillment				0.34 **
Competence need fulfillment				0.24 **
Relatedness need fulfillment				−0.04
R^2^	0.15	0.23	0.08	0.50
F	5.63 **	9.59 **	2.65 *	20.72 **

Note. *N* = 198. Standardized coefficient. * *p* < 0.05, ** *p* < 0.01.

**Table 3:** Indirect effects of advice network centrality on task crafting.

Mediators	Effect (*b*)	SE	LLCI	ULCI
Total indirect effect	0.006	0.002	0.002	0.009
Need for autonomy	0.004	0.002	0.001	0.007
Need for competence	0.002	0.001	0.000	0.004
Need for relatedness	−0.000	0.000	−0.001	0.001

Note. SE = standard error, LLCI = lower limit of the 95% confidence interval, ULCI = upper limit of the 95% confidence interval.

## Data Availability

The data presented in this study are available upon request from the corresponding author.
